# 3D Interconnected V_6_O_13_ Nanosheets Grown on Carbonized Textile via a Seed-Assisted Hydrothermal Process as High-Performance Flexible Cathodes for Lithium-Ion Batteries

**DOI:** 10.1186/s11671-018-2469-6

**Published:** 2018-03-01

**Authors:** Shixing Xu, Dingcheng Cen, Peibo Gao, Huang Tang, Zhihao Bao

**Affiliations:** 1 Shanghai Key Laboratory of Special Artificial Microstructure Materials and Technology, School of Physics Science and Engineering, Tongji University, 1239 Siping Road, Shanghai, 200092 China; 2 School of Mathematics and Physics, Jiangsu University of Technology, 1801 Zhongwu Road, Changzhou, 213001 China

**Keywords:** Interconnected V_6_O_13_ nanosheets, Seed-assisted hydrothermal, Lithium-ion batteries

## Abstract

Three-dimensional (3D) free-standing nanostructured materials have been proven to be one of the most promising electrodes for energy storage due to their enhanced electrochemical performance. And they are also widely studied for the wearable energy storage systems. In this work, interconnected V_6_O_13_ nanosheets were grown on the flexible carbonized textile (c-textile) via a seed-assisted hydrothermal method to form a 3D free-standing electrode for lithium-ion batteries (LIBs). The electrode exhibited a specific capacity of 170 mA h g^−1^ at a specific current of 300 mA g^−1^. With carbon nanotube (CNT) coating, its specific capacities further increased 12–40% at the various current rates. It could retain a reversible capacity of 130 mA h g^−1^, 74% of the initial capacity after 300 cycles at the specific current of 300 mA g^−1^. It outperformed most of the mixed-valence vanadium oxides. The improved electrochemical performance was ascribed to the synergistic effect of the 3D nanostructure of V_6_O_13_ for feasible Li^+^ diffusion and transport and highly conductive hierarchical conductive network formed by CNT and carbon fiber in c-textile.

**Electronic supplementary material:**

The online version of this article (10.1186/s11671-018-2469-6) contains supplementary material, which is available to authorized users.

## Background

Vanadium oxides (e.g., V_6_O_13_, V_3_O_7_, V_2_O_5_) are cathode materials applicable for high-energy lithium-ion battery (LIB), due to their low cost, high specific capacities, and the abundance of vanadium element [1–6]. Among the oxides, V_6_O_13_ has been considered as an excellent candidate of the cathode material [7–14]. Its theoretical capacity and energy density can reach 417 mA h g^−1^ and 890 Wh kg^−1^ when lithiated to the final product, Li_8_V_6_O_13_ [2, 8]. However, V_6_O_13_ electrodes have suffered short cycle life and low rate capability for a long time because V_6_O_13_’s electronic conductivity decreases when lithiated while Li^+^ diffusion coefficients (10^−8^ to 10^−9^ cm^2^ S^−1^) are low [7, 9]. Constructing free-standing 3D nanostructures is an effective method to solve the above problems. 3D nanostructure can enhance ion/electron transport/diffusion while effectively avoids self-aggregation [15–20]. For example, Yu et al. synthesized 3D V_6_O_13_ nanotextiles assembled from interconnected nanogrooves via a facile solution-redox-based self-assembly route with MnO_2_ template at room temperature. In a voltage range of 1–4 V, V_6_O_13_ nanotextiles exhibited reversible capacities of 326 and 134 mA h g^−1^ at 20 and 500 mA g^−1^, respectively, and a capacity retention of above 80% after 100 cycles at 500 mA g^−1^ [2]. Tong et al. fabricated V_6_O_13_ cathode supported by a steel mesh with wrinkles by the similar route. The free-standing electrode with a loading amount of V_6_O_13_ up to 2.0 mg cm^−2^ was obtained. At a current density of 500 mA g^−1^, the V_6_O_13_ electrode demonstrated an initial capacity of 225 mA h g^−1^ that deteriorated to around 150 mA h g^−1^ after 500 cycles [21]. However, above research involved the two-step electrodeposition and removal of MnO_2_. Direct growth of mixed-valence vanadium oxide nanostructure with good electrochemical property remains a great challenge [22]. Meanwhile, previous studies have not demonstrated V_6_O_13_-based flexible cathode, which has a potential use in the wearable devices.

Herein, we proposed a simple hydrothermal process to successfully grow interconnected V_6_O_13_ nanosheets on the carbonized textile to fabricate a 3D free-standing electrode. It exhibited specific capacities of 161 and 105 mA h g^−1^ at the specific currents of 300 and 1200 mA h g^−1^, respectively. With carbon nanotube (CNT) coating to further improve its conductivity, its specific capacities increased to 170 and 140 mA h g^−1^. Meanwhile, its cycling performance was also improved. It could retain 74% of initial capacity with CNT coating, compared with 50% retention without CNT coating after 400 cycles at 300 mA g^−1^. The improvement on the electrochemical performance was mainly ascribed to the synergistic effect of the 3D nanostructure of V_6_O_13_ and hierarchical conductive network.

## Methods

### Synthesis of c-textile

The commercially available bamboo cloth was soaked in a solution with 2.5 g NaF and 60 ml H_2_O for 1 h and dried for 5 h in 120 °C oven. The dried textile was carbonized at 800 °C in N_2_ for 30 min to obtain c-textile.

### Growth of 3D V_6_O_13_ Nanostructure on c-textile

3D V_6_O_13_ nanostructure was grown on c-textile by a seed-assisted hydrothermal method. c-textile was slightly oxidized in the condensed nitric acid (80 wt%) for 30 min. V_2_O_5_ powder (1 mg) was added to 5 ml deionized water and then ultrasonicated for 15 min to obtain a suspension. The oxidized c-textile was then immersed into the suspension for 2 h, dried, and heated at 300 °C for 10 min to grow the seed of vanadium oxide on c-textile. V_2_O_5_ powder (16 mg) was added to 224 μl of 30 wt% H_2_O_2_ and stirred for 10 min to obtain a brown solution. It was then diluted with additional 40 ml distilled water and stirred for 30 min. After the solution was transferred into a 25-ml stainless-steel autoclave, the oxidized c-textile was immersed into the solution. The autoclave was kept at 180 °C for 48 h, then the sample was washed with distilled water and alcohol and dried at 60 °C for 8 h to finally get the flexible 3D free-standing V_6_O_13_ nanostructure supported with flexible c-textile. CNT was further coated on V_6_O_13_ nanostructure by repeatedly dipping it into NMP suspension (0.5 mg/mL) of multi-walled CNT and drying to produce a V_6_O_13_/CNT composite electrode.

### Characterization of Materials

Morphology of the product was observed by a scanning electron microscopy (SEM, Philips XL30 FEG) and a transmission electron microscopy (TEM, JEOL JEM-2010). X-ray photoelectron spectroscopy (XPS) analyses (K-Alpha) were performed using a monochromatic Al Ka source.

### Battery Fabrication and Electrochemical Measurements

Standard CR2016-type coin cells were assembled in an argon-filled glove box (Vigor Inc. Suzhou, China) with V_6_O_13_ electrode as the working electrode with a mass loading of ~ 1 mg cm^−2^. A lithium foil was used as the counter electrode; 1 mol LiPF_6_ in a mixture of ethylene carbonate (EC), diethyl carbonate (DEC), and dimethyl carbonate (DMC) with a volume ratio of 1:1:1 was used as the electrolyte, and a polypropylene film was used as the separator. The assembled cells were electrochemically cycled between 1.5 and 4.0 V vs. Li/Li^+^ for galvanostatic charge/discharge on a LAND battery test system (Wuhan Kingnuo Electronics Co., Ltd., China) at 25 °C. Electrochemical impedance spectroscopy (EIS) studies were conducted with Autolab PGSTAT302N workstation in the frequency range of 10 mHz to 10 kHz.

## Results and Discussion

**Fig. 1:**
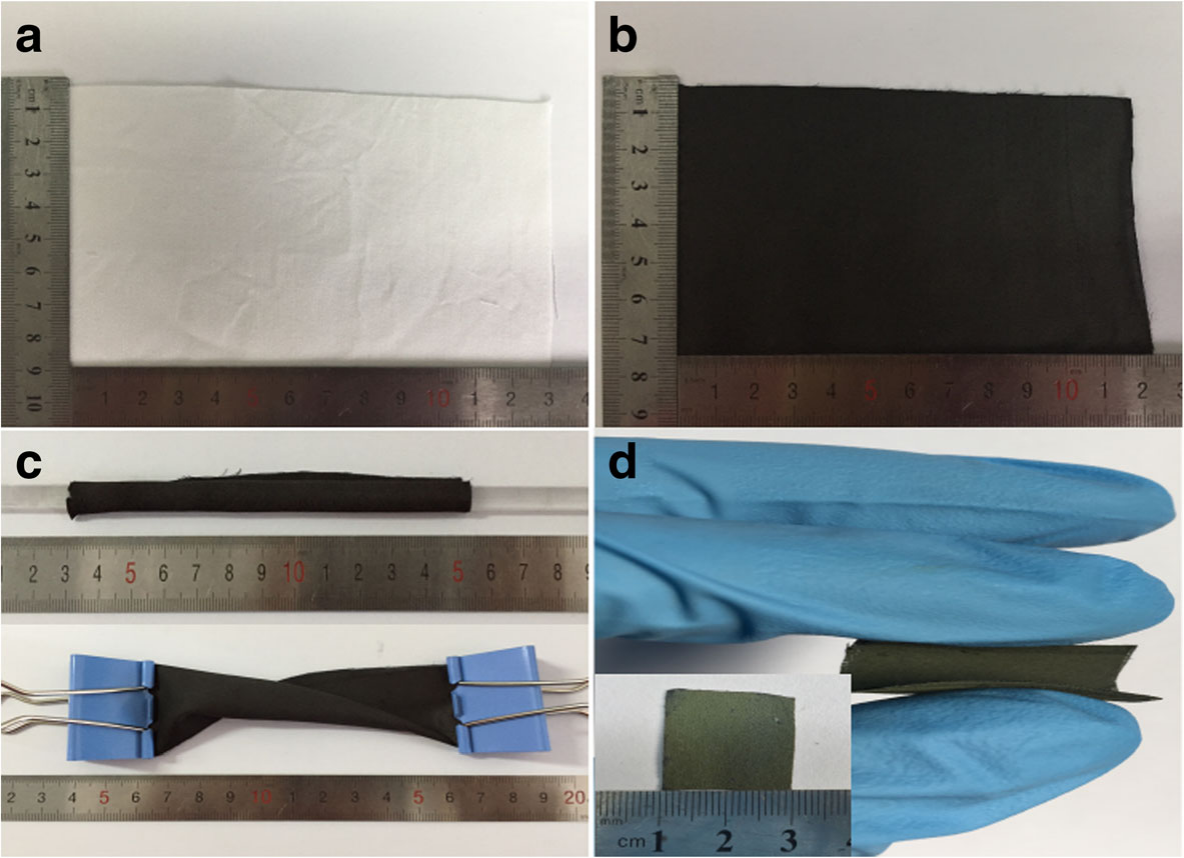
Optical images of **a** commercially available textile, **b** carbonized textile, **c** rolled and twisted c-textile, and **d** c-textile with grown V_6_O_13_ at the rolled state, inset: at the flat state

**Fig. 2:**
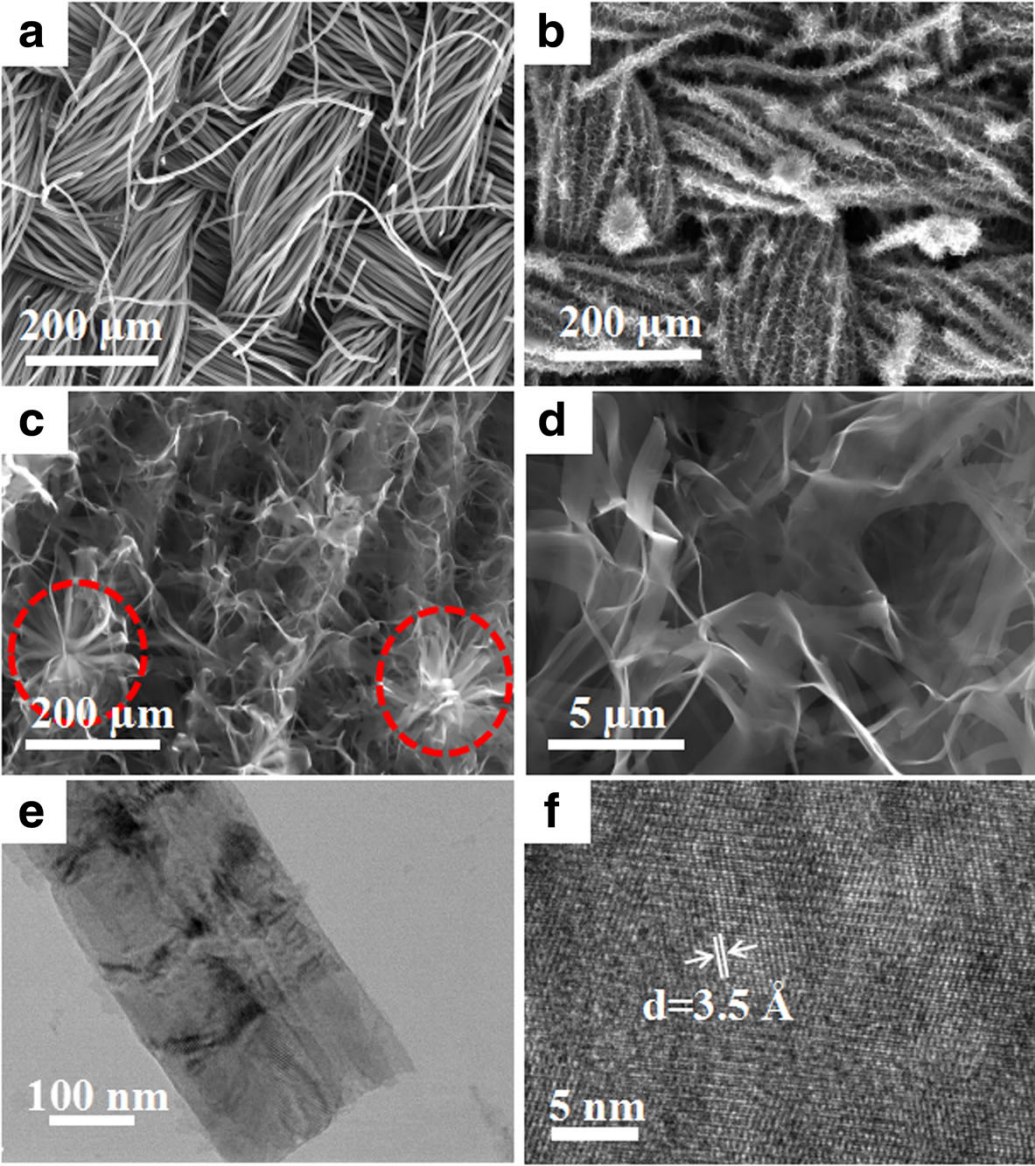
Microstructure of 3D free-standing interconnected V_6_O_13_ nanosheets on c-textile: **a**, **b** low resolution SEM images of c-textile without and with nanosheets, respectively; **c**, **d** high-resolution SEM images of interconnected nanosheets grown on c-textile; **e**, **f** low- and high-resolution TEM images of the nanosheet, respectively

**Fig. 3:**
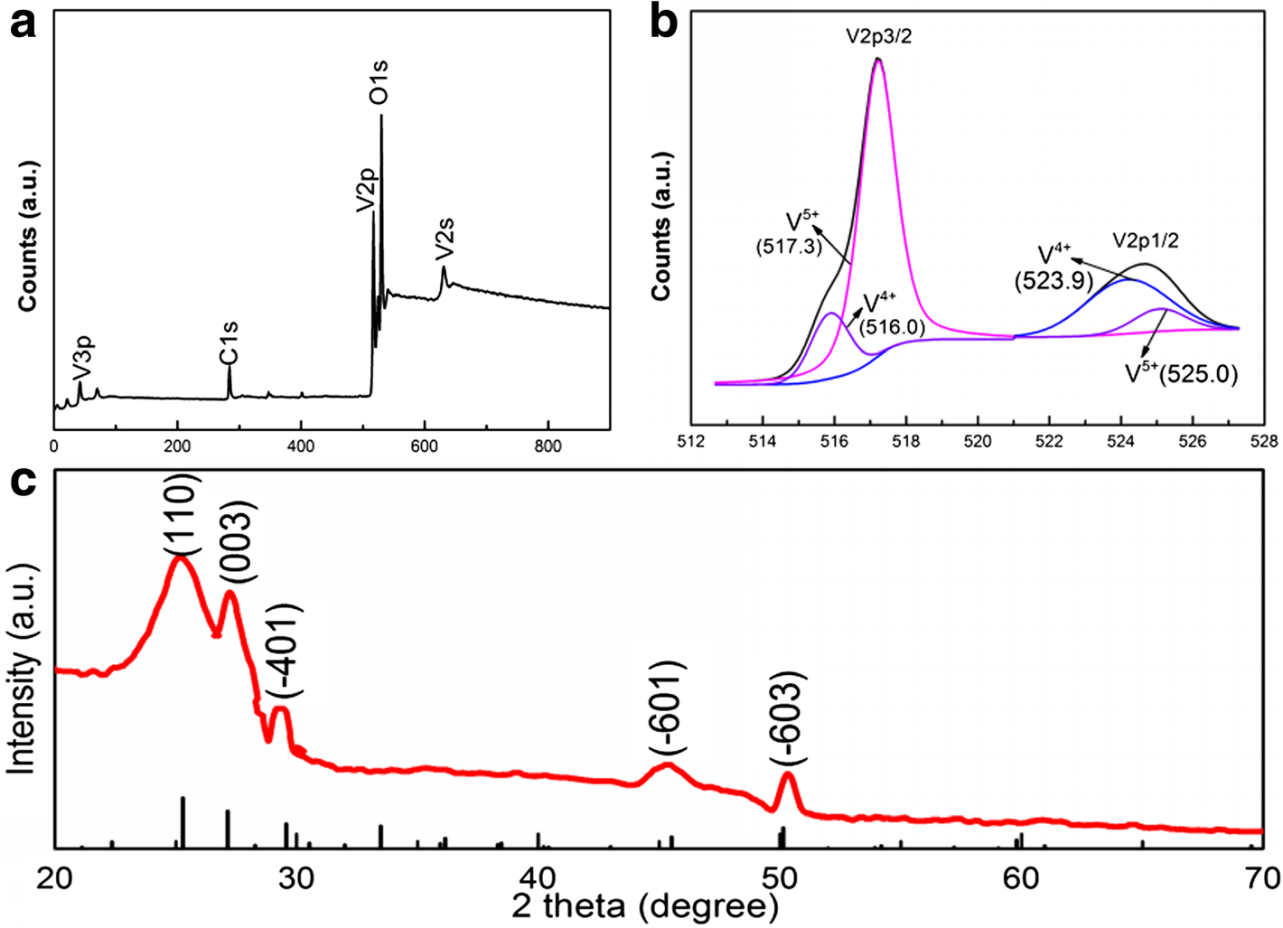
**a** A survey XPS spectrum of 3D free-standing interconnected V_6_O_13_ nanosheets grown on c-textile. **b** Spectrum of V_2p_ and O_1s_ with a fitted curve. **c** The XRD patterns of V_6_O_13_

The schematic of the growth of 3D V_6_O_13_ interconnected nanosheets on c-textile was shown in Additional file 1: Figure S1. The textile (Fig. 1a) was firstly carbonized at 800 °C to obtain c-textile (Fig. 1b). SEM images (Fig. 2a) showed that c-textile was composed of weaved bundles of carbon fibers with a diameter of ~ 5 μm. c-textile exhibited excellent flexibility and mechanical strength. It was able to be rolled and twisted as shown in Fig. 1c. The square resistivity of the c-textile was measured to be 5 Ω/sq. with the four-probe method. Thus, it was used as a promising flexible support/collector for the electrode materials. It was then immersed in VO_x_ suspension, dried, and kept at 300 °C for 10 min to grow the seed crystals. Its weight change was undetectable (< 0.1 mg). After being immersed in vanadium oxide (VO_x_) sol solution for the hydrothermal growth, the black c-textile was covered with a layer of a yellow-green thin film; however, its flexibility was kept, as shown in Fig. 1d. Its resistivity increased to 50 Ω/sq. SEM images (Fig. 2b, c) further showed that it was composed of several micron-long and several hundred nanometer-wide interconnected nanosheets, as building blocks to construct 3D nanostructure on c-textile. High-resolution TEM image (Fig. 2f) showed well-defined lattice fringes of the grown nanosheets. The spacing of 3.5 Å in the lattice fringe was consistent with (110) interplanar distance of the orthogonal V_6_O_13_ phase (PDF card No.71-2235) which was in agreement with the XRD pattern (Fig. 3c). The growth mechanism was that seed crystal firstly nucleated on the sites with an oxygen-bearing functional group [23, 24]. Then during the hydrothermal process in the VO_x_ aqueous solution, interconnected V_6_O_13_ nanosheets were continuously grown on the seed crystals. As for the formation of 3D structured microflowers, it might be due to the several seed crystals aggregated at the same location for growth of the nanosheets. To further determine the valence state of vanadium element in V_6_O_13_, XPS analyses were conducted on the synthesized interconnected V_6_O_13_ nanosheets. The survey XPS scan (Fig. 3a) revealed that the sample was composed of V, O, C, and N elements. The binding energies for vanadium 2p3/2 and 2p1/2 were identified in Fig. 3b at 516.0 and 523.9 eV for V^4+^ and 517.3 and 525.0 eV for V^5+^, respectively. It was well consistent with the chemical state of vanadium in V_6_O_13_ reported [25–27]. The above results confirmed that 3D V_6_O_13_ nanostructures were successfully grown on c-textile via a simple seed-assisted hydrothermal process.

**Fig. 4:**
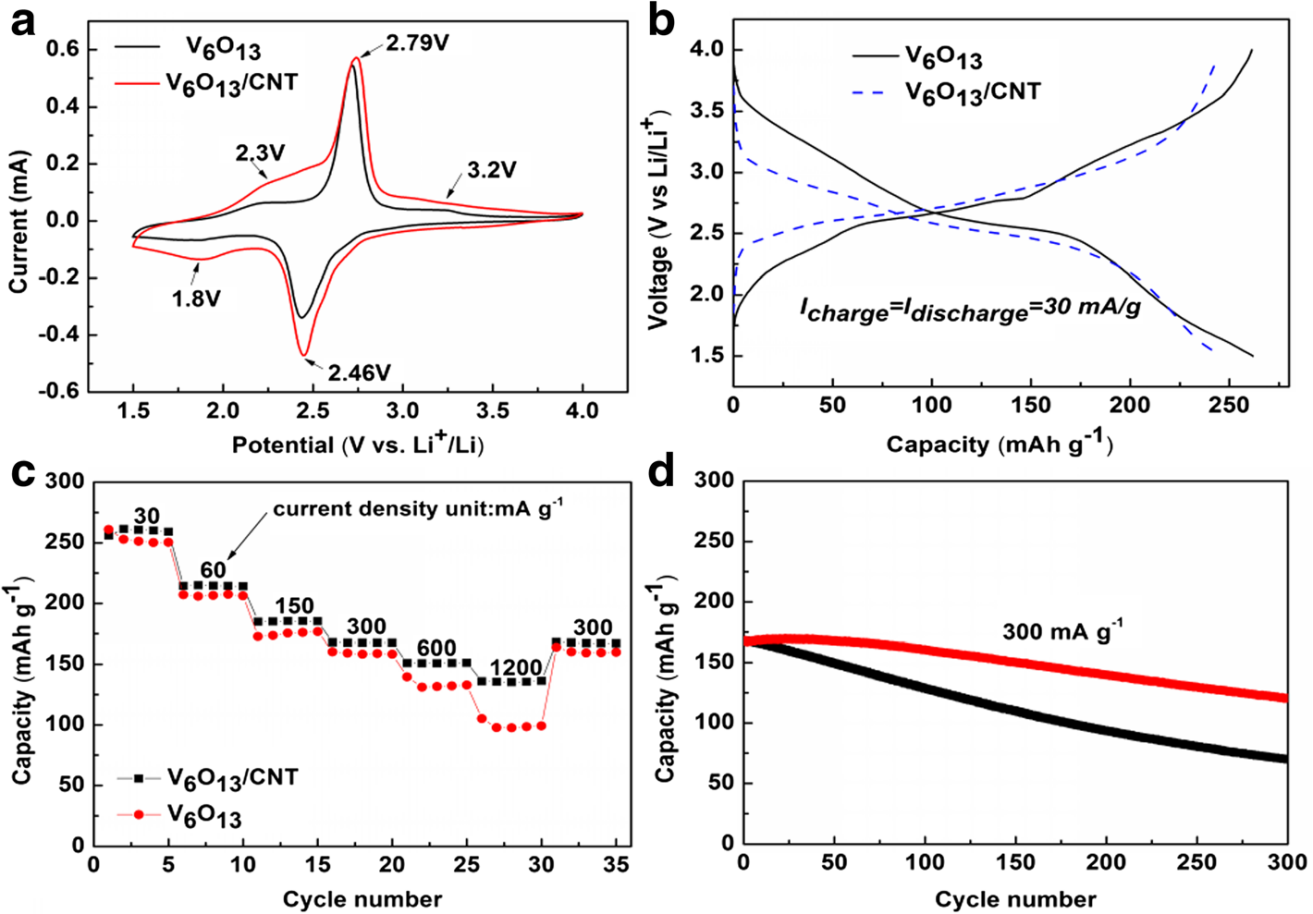
Electrochemical performance of 3D free-standing V_6_O_13_ electrodes with/without CNT coating. **a** Cyclic voltammetry curves. **b** Galvanostatic charge/discharge curves. **c** Rate. **d** Cyclability performance of the two electrodes

**Fig. 5:**
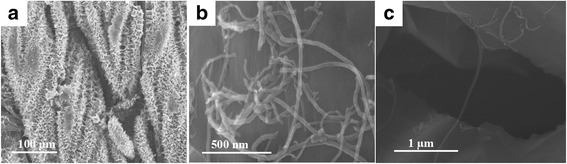
Microstructure of CNT-coated V_6_O_13_ electrode. **a** Low-resolution SEM image of the electrode. **b**, **c** High-resolution SEM image of the electrode showing CNT covering on the nanosheet and bridging between the nanosheets

**Fig. 6:**
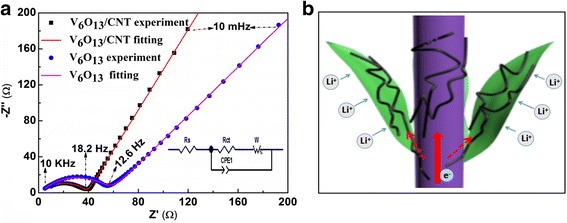
**a** Nyquist plots of V_6_O_13_ electrode with/without CNT coating. **b** Schematic of the transporting path for electrons in CNT-coated V_6_O_13_ electrode

To investigate the electrochemical performance of 3D V_6_O_13_ nanostructures grown on c-textile, half-cell coin batteries were assembled with a V_6_O_13_ electrode as the cathode and lithium foil as the anode. Figure 4a displayed typical cyclic voltammetry (CV) curves of V_6_O_13_ electrode in a scan rate of 0.2 mV s^−1^ between 1.5 and 4.0 V (vs. Li/Li^+^). The main redox peaks at 2.8/2.5 V could be easily identified. Broad anodic peak at ~ 3.2 and 2.3 V and cathodic peak at ~ 1.8 V could also be observed [11, 28]. The appearance of the above peaks indicated multi-step phase transitions, and the location of peak voltages was consistent with the previously reported ones [2]. Figure 4b showed the galvanostatic discharge/charge curve for the first cycle at the specific current of 30 mA g^−1^. Multiple poorly defined voltage plateaus could be identified. In the discharge curve, two sloped plateaus were identified at 2.3 and 2.8 V, corresponding to the anodic peaks. When the specific currents increased from 30 to 150, 300, 600, and 1200 mA g^−1^, the specific capacities were 253, 176, 161, 133, and 105 mA h g^−1^. The good electrochemical properties were due to the 3D nanostructure composed of V_6_O_13_ nanosheets. Such open structure could contact with electrolytes very well and shorten the Li^+^ transport and diffusion path. The morphology of the original V_6_O_13_ electrode and the cycled electrode with SEI was examined under SEM (Additional file 1: Figure S2). The morphology of 3D interconnected nanosheets was retained during the cycling. This further suggests the importance of the 3D nanostructure on the structural integrity of V_6_O_13_ electrode during the electrochemical cycling. However, the conductivity of V_6_O_13_ decreased as lithiation proceeded [7, 29]. Moreover, the length of nanosheet in the V_6_O_13_ electrode reached several tens of microns. Only a small portion of individual nanosheet is directly connected with the conductive carbon fibers of c-textile, which served as the collector. It could hinder the electron transfer during the charge/discharge process. To further enhance the conductivity, and thus the electrochemical properties of the 3D free-standing V_6_O_13_ electrode, it was immersed in CNT dispersion to dip-coat CNT on its surface. Figure 5a, b showed SEM images of the V_6_O_13_ electrode with CNT. CNT was successfully deposited in the plane of V_6_O_13_ nanosheets and intimately contacted with them. Even the bridging was built through CNTs between neighboring nanosheets, as shown in Fig. 5c. As expected, the resistivity of the V_6_O_13_ electrode with CNT decreased from 50 to 20 Ω/sq. After the coating of CNTs, the redox peak appeared at the same position on CV profile (Fig. 4a) while peak currents increased. It indicated fast kinetic of electrochemical reaction in V_6_O_13_ electrode with CNT. The V_6_O_13_ electrode with CNT exhibited better rate performance compared with the electrode without CNT coating, as shown in Fig. 4c. The specific discharge capacities were 261, 185, 170, 153, and 140 mA h g^−1^ at the specific currents of 30, 150, 300, 600, and 1200 mA g^−1^, respectively, corresponding to 12~40% increase compared with the composite cathode without CNTs. To further verify the role of CNT, we calculated the lithium ion diffusion coefficient with cyclic voltammetry. The V_6_O_13_/CNT anodic and cathodic diffusion coefficients were 4.79 × 10^−8^ and 2.01 × 10^−8^ cm^2^ s^−1^, higher than V_6_O_13_ electrode’s 2.42 × 10^−8^ and 1.7 × 10^−8^ cm^2^ s^−1^, respectively (and the associated discussion is in Additional file 1: Figure S3). Nyquist plots (Fig. 6a) of V_6_O_13_ electrode and V_6_O_13_ electrode with CNT displayed similar shapes, a semicircle shape in the high-to-medium frequency domain and an inclined line in the low-frequency regions, corresponding to electrochemical reaction impedance (charge transfer process) and diffusion process of lithium ions. The inset is the equivalent circuit used to fit Nyquist plots. In the circuit, CPE is the constant phase angle element and W is the Warburg impedance. *R*_s_ and *R*_ct_ represent the ohmic resistance (total resistance of the electrolyte, separator, and electrical contacts) and the charge transfer resistance, respectively [22, 30]. Additional file 1: Table S1 listed the parameters used to fit the plots. *R*_ct_ for the V_6_O_13_/CNT electrode was calculated to be 37.24 Ω, lower than that of V_6_O_13_ (55.58 Ω). This decrease in charge transfer resistance was ascribed to the addition of CNT. The mechanism was illustrated in Fig. 6b. CNT intimately connected with V_6_O_13_ nanosheets for faster electron transferring. Furthermore, CNTs and carbon fiber in the c-textile composed hierarchical conductive network for better electron conducting. The cyclability of V_6_O_13_ electrodes was shown in Fig. 4d. At the specific current of 300 mA g^−1^, the electrode with CNT coating could maintain 74% of the initial capacity of 170 mA h g^−1^ after 300 charge/discharge cycles, while the V_6_O_13_ electrode only retained 42% of its initial capacity. It outperformed most of the low dimensional mixed-valence vanadium oxides or their 3D nanostructure listed in Additional file 1: Table S2. The better cyclability of V_6_O_13_ electrode with CNT might be ascribed to the following reasons: (1) Reinforced with CNT, V_6_O_13_’s mechanical properties were improved. (2) Even if V_6_O_13_ nanostructure was broken during the discharge/charge process, it was still attached to CNT and could be electrochemically activated. (3) Self-segregation of V_6_O_13_ nanosheets was limited by the appearance of CNT. (4) CNT coating might be a valid barrier to alleviate side reaction of vanadium oxide with electrolyte, if any. Thus, CNT coating can be a facile alternative way to improve the conductivity of the 3D nanostructure, other than carbon coating and polymeric coating which usually require tremendous chemical synthesis work [14]. The overall electrochemical performance of V_6_O_13_ cathode was limited by the conductivity of carbon cloth, the Li diffusivity in V_6_O_13_ materials, and electron transfer between V_6_O_13_ nanostructures and the carbon cloth. In the future work, further improvement can be made in the following ways: (1) reducing the resistance of the carbon cloth substrate, (2) doping V_6_O_13_ with sulfur to improve its diffusivity of lithium ion, and (3) coating the V_6_O_13_ with conductive polymer coating.

## Conclusions

In summary, we successfully fabricated 3D free-standing V_6_O_13_ nanostructure composed of interconnected nanosheets via a facile seed-assisted hydrothermal process as a cathode for LIB. The electrode exhibited good electrochemical performance. It could be further improved by coating 3D V_6_O_13_ nanostructure with CNT, outperforming most of the mixed-valence vanadium oxides. Its excellent performance was due to its open 3D nanostructure and hierarchical conductive network composed of CNT in nanoscale and carbon fiber in microscale. The design of 3D nanostructure with the building block (e.g., nanowire, nanosheet) combined with constructing of the hierarchical conductive path by CNT coating can be extended to other electrode materials for better electrochemical performance.

## Additional file

Additional file 1: 3D interconnected V_6_O_13_ nanosheets grown on carbonized textile via a seed-assisted hydrothermal process as high-performance flexible cathodes for lithium-ion batteries. (DOCX 1386 kb)

## Abbreviations

3D: Three dimensional
CE: Coulombic efficiency
CNT: Carbon nanotube
c-textile: Carbonized textile
CV: Cyclic voltammetry
DEC: Diethyl carbonate
DMC: Dimethyl carbonate
EC: Ethylene carbonate
EIS: Electrochemical impedance spectroscopy
LIB: Lithium-ion battery
SEM: Scanning electron microscopy
TEM: Transmission electron microscopy
