# Supplementary material for: 3D Interconnected V6O13 Nanosheets Grown on Carbonized Textile via a Seed-Assisted Hydrothermal Process as High-Performance Flexible Cathodes for Lithium-Ion Batteries
Source: Nanoscale Res Lett. 2018 Mar 1;13:65. doi: 10.1186/s11671-018-2469-6 (PMC5834947; doi:10.1186/s11671-018-2469-6)
Supplement: Supplementary file 1 — 3D interconnected V6O13 nanosheets grown on carbonized textile via a seed-assisted hydrothermal process as high-performance flexible cathodes for lithium-ion batteries. (DOCX 1386 kb) [file 11671_2018_2469_MOESM1_ESM.docx]

Additional file

3D interconnected V_6_O_13_ nanosheets grown on carbonized textile via a seed-assisted hydrothermal process as high-performance flexible cathodes for lithium ion batteries

Shixing Xu, Dingcheng Cen, PeiBo Gao, HuangTang, and Zhihao Bao


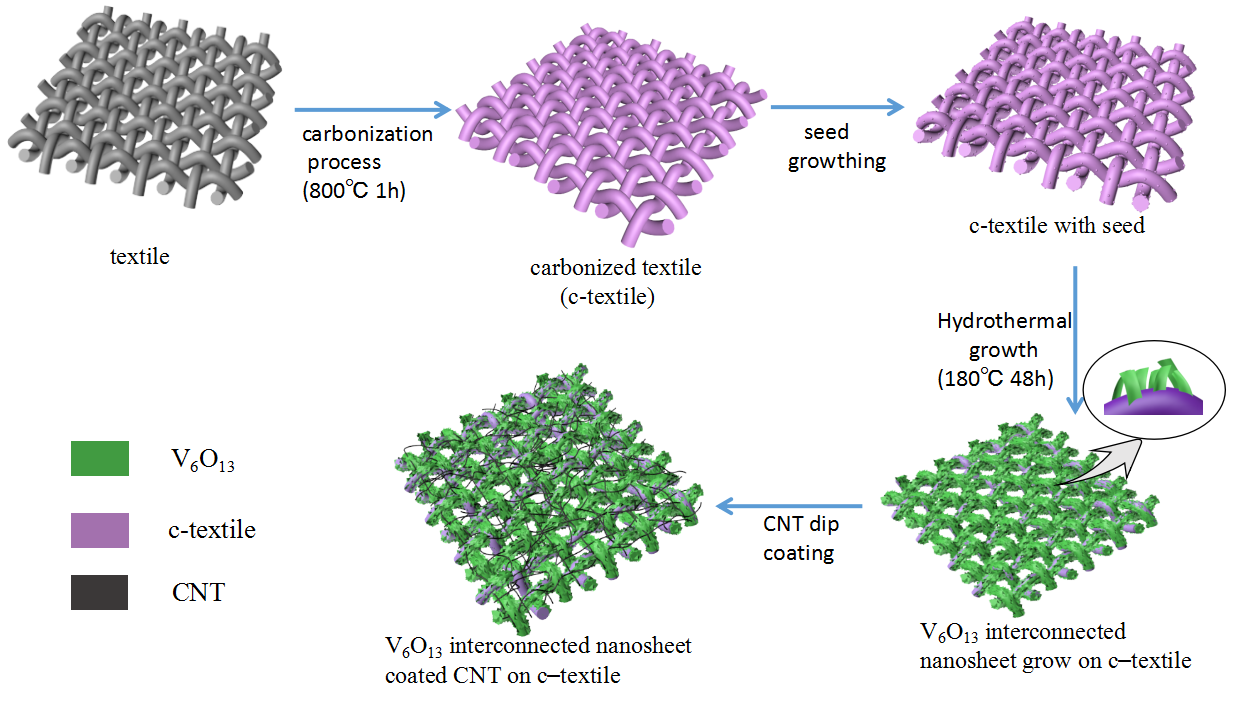


Figure S1: Schematic diagram of the synthesis of 3D free-standing interconnected V_6_O_13_ nanosheets on the flexible carbonized textile.


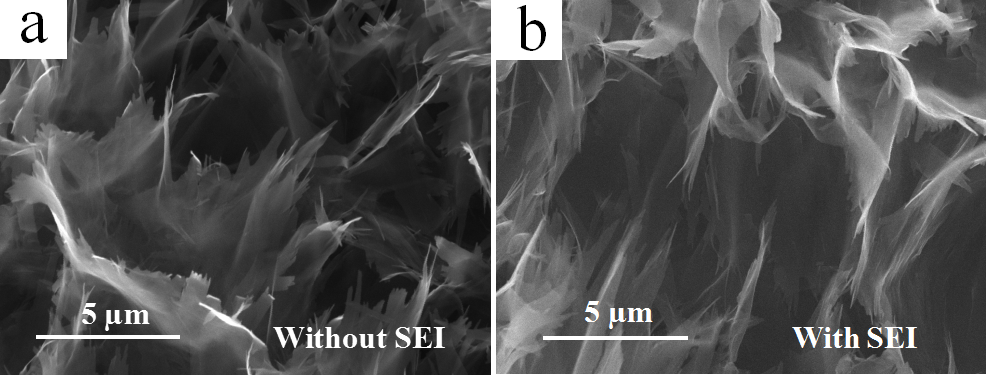


Figure S2 Typical SEM images of nanosheets (a) before and (b) after 20 cycles. The morphology of V_6_O_13_ nanosheets with SEI was largely unchanged compared to those of the original V_6_O_13_.


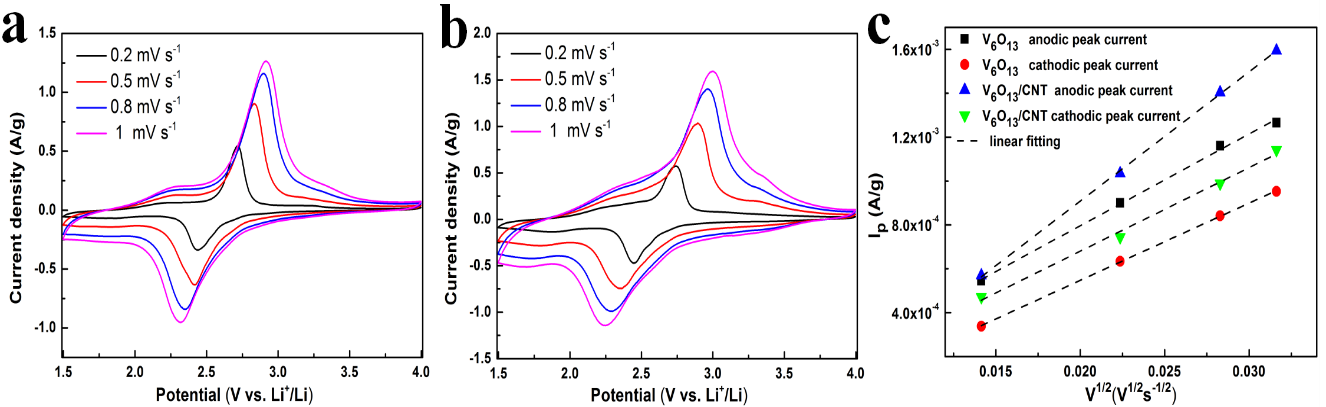


Figure S3 CV curves at different scan rates of (a) V_6_O_13_ and (b) V_6_O_13_/CNT (C) The corresponding relationship between the square root of the scan rate v^1/2^ and peak current I_p_ of V_6_O_13_ and V_6_O_13_/CNT.

In order to further study the Li^+^ ion diffusion coefficients of V_6_O_13_ and V_6_O_13_/CNT, CV curves of the half batteries at various scan rates are displayed in Fig. S3a,3b. The anodic peaks shift to low potential and the cathodic peaks shift to high potential with increasing scan rate. The peak current increases with increasing rate, and the V_6_O_13_/CNT electrode peak currents are slightly higher than the V_6_O_13_ electrode peaks, indicating slightly lithium ion diffusion difference. As can be seen from Fig S3c, the peak current (Ip) shows a linear relation with the square root of the sweep rate (v^1/2^). This is a typical diffusion controlling response, the chemical diffusion coefficient (D_Li_^+^) can be calculated from the Randles-Sevcik equation[1][2]:

I_p_ = 0.4463n^3/2^F^3/2^CSR^−1/2^T^−1/2^D^1/2^ v^1/2^

Where Ip is the peak current (A), n is the charge transfer number, F is the Faraday’s constant (8.314 J mol^-1^ K^-1^), T is the absolute temperature (K), D is the chemical diffusion coefficient (cm^2^s^-1^), and v is the scanning rate (V s^-1^).Herein the chemical diffusion coefficients of lithium can be calculated by Eq. (1) from the slope of d(I_p_)/d(v^1/2^), which are 2.42×10^-8^ cm^2^ s^-1^ and 1.7×10^-8^ cm^2^ s^-1^ for the V_6_O_13_ anodic oxidation and cathodic reduction process ,respectively, the V_6_O_13_/CNT diffusion coefficient of anode and cathode are 4.79×10^-8^ cm^2^ s^-1^ and 2.01×10^-8^ cm^2^ s^-1^. It’s clear that the D value of V_6_O_13_/CNT is higher than that of V_6_O_13_ which should attributed to the CNT. The coating of carbon nanotubes increases the diffusion rate of lithium ions in the active materials

Table S1. The parameters of equivalent circuit used to fit Nyquist plot of V_6_O_13_ electrodes with/without CNT coating.

| Fitted results | | | | | | | |
| --- | --- | --- | --- | --- | --- | --- | --- |
|  | R_s_ | R_ct_ | W-R | W-T | W-P | CPE-T | CPE-P |
| V_6_O_13_/CNT | 2.169 | 37.24 | 3011 | 126.3 | 0.73511 | 6.27×10^-5^ | 0.65077 |
| V_6_O_13_ | 2.172 | 55.58 | 1347 | 189.3 | 0.59532 | 1.11×10^-4^ | 0.68137 |

Table S2. List of mixed valence VO_x_ nanostructure with their electrochemical performance.

| Electrode description | Specific capacity (mAh/g) | Cycling stability (%) | Voltage range | Reference |
| --- | --- | --- | --- | --- |
| 3D freestanding V_6_O_13_ | 170 mAh/g at 300 mA/g | 96.7% after 100 cycles 76% after 300 cycles at 300 mA/g | 1.5-4 V | This work |
| Single crystal V_3_O_7_·H_2_O | 290 mAh/g at 1 A/g | 51.6% after 30cycles at 20 mA/g | 1.5-3.75 V | [3] |
| V_3_O_7_·H_2_O nanobelts | 373 mAh/g at 0.2 mA/cm^2^ | 28.2% after 30 cycleat 0.2 mA/cm^2^ | 1.5-4 V | [4] |
| VO_x_-NT | 225 mAh/g at 100 mA/g | 15.7% after 50 cycles at 50 mA/g | 1.5-4 V | [5] |
| V_3_O_7_·H_2_O nanobelts | 253 mAh/g at 30 mA/g | 90% after 56 cycles at 30 mA/g | 1.5-4 V | [6] |
| V_3_O_7_@C composites | 200 mAh/g at 0.2 mA/cm^2^ | 75.5% after 45 cycles at 0.2 mA/cm^2^ | 1.5-4 V | [7] |
| PEO/ V_3_O_7_·nH_2_O | 297 mAh/g at 24.75 mA/cm^2^ | 68% after 30 cycles at 24.75 mA/cm^2^ | 2-4 V | [8] |
| Fe-VO_x_-NTs | 311 mAh/g at 50 mA/g | 57.2% after 50 cycles at 50 mA/g | 1.5-4 V | [9] |
| 3D V_6_O_13_ nanotextiles | ~215 mAh/g at 200 mA/g | 80% after 100 cycles at 500 mA/g | 1.5-4 V | [10] |
| Steel-mesh-supported V_6_O_13_ | ~170 mAh/g at 500 mA/g | 53% after 500 cycles at 500 mA/g | 1.5-4 V | [11] |
| V_6_O_13_(A2-160) nanosheets | 330 mAh/g at 42 mA/g | 72% after 50 cycles at 42 mA/g | 1.5-3.5 V | [12] |

References:

1 Xu N, Ma X, Wang M, et al. Stationary Full Li-Ion Batteries with Interlayer-Expanded V_6_ O_13_ Cathodes and Lithiated Graphite Anodes[J]. Electrochimica Acta, 2016, 203: 171-177.

2 Tang K, Yu X, Sun J, et al. Kinetic analysis on LiFePO_4_ thin films by CV, GITT, and EIS[J]. Electrochimica Acta, 2011, 56(13): 4869-4875.

3 Gao, S.; Chen, Z.; Wei, M.; Wei, K.; Zhou, H. Single crystal nanobelts of V_3_O_7_·H_2_O: A lithium intercalation host with a large capacity. Electrochimica Acta 2009, *54*, 1115-1118.

4 Zhang, Y.; Liu, X.; Xie, G.; Yu, L.; Yi, S.; Hu, M.; Huang, C. Hydrothermal synthesis, characterization, formation mechanism and electrochemical property of V_3_O_7_·H_2_O single-crystal nanobelts. Materials Science and Engineering*: B* 2010, *175*, 164-171.

5 Zhou, X.; Cui, C.; Wu, G.; Yang, H.; Wu, J.; Wang, J.; Gao, G. A novel and facile way to synthesize vanadium pentoxide nanospike as cathode materials for high performance lithium ion batteries. Journal of Power Sources 2013, *238*, 95-102.

6 Qiao, H.; Zhu, X.; Zheng, Z.; Liu, L.; Zhang, L. Synthesis of V_3_O_7_·H_2_O nanobelts as cathode materials for lithium–ion batteries. Electrochem Commun 2006, *8*, 21-26.

7 Zhang, Y.; Zhou, M.; Fan, M.; Huang, C.; Chen, C.; Cao, Y.; Li, H.; Liu, X. Improvement of the electrochemical properties of V_3_O_7_·H_2_O nanobelts for li battery application through synthesis of V_3_O_7_@C core-shell nanostructured composites. Current Applied Physics 2011, *11*, 1159-1163.

8 Mohan, V. M.; Murakami, K.; Chen, W. The influence of peo on the synthesis and electrochemical properties of VO_2_ and V_3_O_7_·nH_2_O nanobelts as a cathode for lithium battery. Ionics 2012, *18*, 607-614.

9 Zhou, X.; Wu, G.; Gao, G.; Wang, J.; Yang, H.; Wu, J.; Shen, J.; Zhou, B.; Zhang, Z. Electrochemical performance improvement of vanadium oxide nanotubes as cathode materials for lithium ion batteries through ferric ion exchange technique. The Journal of Physical Chemistry *C* 2012, *116*, 21685-21692.

10 Ding, Y. L.; Wen, Y.; Wu, C.; van Aken, P. A.; Maier, J.; Yu, Y. 3D V_6_O_13_ nanotextiles assembled from interconnected nanogrooves as cathode materials for high-energy lithium ion batteries. Nano letters 2015, *15*, 1388-94.

11 Wu, Z.; Qiu, W.; Chen, Y.; Luo, Y.; Huang, Y.; Lei, Q.; Guo, S.; Liu, P.; Balogun, M.-S.; Tong, Y. Etched current collector-guided creation of wrinkles in steel-mesh-supported V_6_O_13_ cathode for lithium-ion batteries. J. Mater. Chem. *A* 2017, *5*, 756-764.

12 Zou, Z.; Cheng, H.; He, J.; Long, F.; Wu, Y.; Yan, Z.; Chen, H. V_6_O_13_ nanosheets synthesized from ethanol-aqueous solutions as high energy cathode material for lithium-ion batteries. Electrochimica Acta 2014, *135*, 175-180.
